# Exploring Net Immunosuppressive Status with Torque Teno Virus Viral Load in Kidney Transplant Recipients with High Molecular Injury

**DOI:** 10.3390/jcm14072417

**Published:** 2025-04-01

**Authors:** Emilio Rodrigo, Elena González-López, Javier Gonzalo Ocejo-Vinyals, Enrique Pasache, Cristina García-Majado, Covadonga López del Moral, Ana García-Santiago, Adalberto Benito-Hernández, María Victoria Francia, Juan Carlos Ruiz

**Affiliations:** 1 Immunopathology Group, Nephrology Department, Marqués de Valdecilla University Hospital-IDIVAL, University of Cantabria, 39005 Santander, Spainjuancarlos.ruiz@scsalud.es (J.C.R.); 2 Immunopathology Group, Immunology Department, Marqués de Valdecilla University Hospital-IDIVAL, University of Cantabria, 39005 Santander, Spain; egonzalezlope@saludcastillayleon.es (E.G.-L.); javiergonzalo.ocejo@scsalud.es (J.G.O.-V.); 3 Infectious Diseases and Clinical Microbiology Group, Marqués de Valdecilla University Hospital-IDIVAL, University of Cantabria, 39005 Santander, Spain

**Keywords:** antibody-mediated rejection, donor-derived cell-free DNA, immune response, infection, kidney transplantation, monitoring, Torque Teno virus

## Abstract

**Background/Objectives**: New monitoring methods are being developed to improve the kidney transplant outcome. Among them, the measurement of Torque Teno virus load (TTV load) has been associated with the overall immunosuppressive status and the percentage of donor-derived circulating free DNA (dd-cfDNA) with molecular graft injury, mainly related to antibody-mediated rejection (AbMR). Both methods provide complementary information, but they have not been previously used together for the monitoring of kidney transplant recipients (KTx). **Methods**: A prospective study including 42 KTx performed in our centre was conducted, in which we monitored dd-cfDNA using a targeted NGS assay (AlloSeq cfDNA) in the first month and the TTV load with in-house PCR in the first and third months. **Results**: Eleven KTx with high molecular injury defined by dd-cfDNA ≥ 1.0% were selected. The TTV load showed a non-significant trend of being lower in AbMR patients (2.91, IQR 4.18 vs. 3.48, IQR 1.47 log10 copies/mL, *p* = 0.788). No overimmunosuppressed patient developed AbMR, whereas 40% of non-overimmunosuppressed patients showed AbMR (*p* = 0.428). The TTV load increased more in the AbMR-treated KTx (0.00, IQR 4.71 vs. +6.58, IQR 4.04 log10 copies/mL, *p* = 0.042) from months one to three, with all AbMR-treated KTx becoming overimmunosuppressed. KTx with opportunistic infections showed higher TTV loads in the third month (5.18, IQR 5.92 vs. 11.53, IQR 3.54 log10 copies/mL, *p* = 0.024). **Conclusions**: KTx with molecular injury secondary to rejection tended to be less immunosuppressed, as indicated by a low TTV load. After AbMR therapy, all KTx became overimmunosuppressed and suffered a higher risk of opportunistic infections. Dual monitoring provides useful complementary information for the follow-up of kidney transplant recipients.

## 1. Introduction

In the monitoring of kidney transplant recipients, physicians attempt to hold patients at an optimal level of immunosuppression to avoid both the risks associated with overimmunosuppression, mainly infections and cancer, and the risk of triggering an alloimmune response and subsequent rejection if the level of immunosuppression is low [1,2,3]. Along with monitoring certain levels through laboratory tests, such as renal function and albuminuria, and tracking the appearances and titres of newly introduced donor-specific antibodies, the usual practice is to monitor trough blood levels of calcineurin-lowering drugs, attempting to hold them within a narrow therapeutic range. However, it is known that the levels of calcineurin inhibitors are more related to the risk of presenting side effects than the risk of rejection [4]. Some centres perform follow-up biopsies to better identify the status of the renal graft, but this is an invasive technique with potential risks [5]. Hence, it is of great interest to develop new non-invasive monitoring tools that will allow for improved knowledge about the immunological statuses of kidney transplant patients.

In recent years, two new methods of monitoring solid organ transplants have been developed. First, the net immunosuppressive state can be estimated by measuring Torque Teno virus (TTV) levels in the blood. TTV is a member of the Anelloviridae family, which is ubiquitous and non-pathogenic to humans [6]. High TTV loads are associated with a higher risk of infection and other immunosuppressive side effects, whereas low TTV loads are associated with a higher risk of acute rejection in kidney and other solid organ transplants [7,8]. The Vienna group proposed using cut-off values <4.6 log10 c/mL and >6.6 log10 copies/mL to identify kidney transplant recipients with under- and overinmunosuppressive statuses [6]. Elsewhere, the percentage of donor-derived cell-free DNA (dd-cfDNA) has been proposed as a potential biomarker of acute rejection and graft failure in kidney transplantation [9]. Elevated dd-cfDNA levels have been associated with an increased risk of renal graft damage, specifically polyomavirus nephropathy, acute cellular rejection, and, primarily, antibody-mediated rejection (AbMR) [10,11,12,13]. A percentage of dd-cfDNA above 1% is highly suggestive of AbMR, allowing the identification of the group of patients with high molecular damage of the graft [14]. In a previous study, we used dd-cfDNA measurement to identify kidney transplant recipients with greater molecular damage and, therefore, a higher risk of rejection or infection in the kidney graft [12]. The aim of the present study was to analyse whether the simultaneous measurement of the TTV viral load allows us to differentiate which of these patients had underimmunosuppression (acute rejection risk) or overimmunosuppression (infection risk) in order to identify the causes of the molecular graft injury in a non-invasive manner.

## 2. Materials and Methods

We conducted a prospective study including the kidney transplants performed in University Hospital Marqués de Valdecilla from January 2021 to April 2022. All participants were adults and gave written informed consent before undergoing kidney transplantation, which was performed in accordance with the tenets of the Declaration of Helsinki and with the approval of the Regional Ethics Committee of our institution (reference number: PI20/01710). Patients who had received non-kidney solid organ transplants, those from uncontrolled cardiac death donations or from living donors, those with pre-formed donor-specific antibodies, and those with a panel-reactive antibody level greater than 98% were excluded.

Relevant information on the characteristics of the recipient, the donor, and the graft was collected. All acute rejection episodes were confirmed by biopsy. Indicative biopsies were performed if the creatinine level increased by 25% or more from the previous value or if proteinuria persisted at >1 g per day. We considered opportunistic infections to be those associated with cellular and humoral immunosuppression, including viral (CMV, EBV, HSV, VZV, BK polyomavirus-associated nephropathy and recurrent hepatitis, HBV, HCV), bacterial (typical and atypical mycobacteria, others such as Nocardia, Listeria, etc.), and fungal (Pneumocystis jirovecii pneumonia, Candida, and invasive fungal infections) infections. The detection of viral, bacterial, and fungal infections was accomplished through the utilisation of standard microbiological techniques.

Maintenance immunosuppressive therapy consisted of twice-daily tacrolimus, mycophenolate mofetil, and prednisone. Recipients at risk of delayed graft function or/and receiving a graft from an expanded criteria donor were treated with Basiliximab. Kidney transplant recipients with a higher rejection risk due to hypersensitisation or previous graft loss due to acute rejection received induction with thymoglobulin. All patients were administered trimethoprim-sulfamethoxazole for six months as prophylaxis against Pneumocystis jirovecii pneumonia. Valganciclovir was used for three months in CMV IgG-negative recipients of a CMV IgG-positive donor and in those patients who received induction with thymoglobulin.

Plasma tacrolimus concentrations (µg/L) were measured by chemiluminescent microparticle immunoassay using the Architect iSystem (CMIA; Abbott Laboratories, Abbott Park, IL, USA). Trough tacrolimus blood concentrations were adjusted to be between 8 and 12 ng/mL until the third month. Trough mycophenolic acid (MPA) concentrations (mg/L) were quantified by homogeneous enzyme-linked immunosorbent assay (Emit 2000 Mycophenolic Acid Assay; Siemens, Munich, Germany) at month 1. Samples for TTV load measurement were obtained before transplantation and at days 30 and 90 post-transplantation. TTV viral load was determined in duplicate using a recently validated protocol with the commercial TTV R-GENE^®^ kit (bioMérieux, Craponne, France), as previously reported [15,16]. Under- and overimmunosuppressive statuses were defined according to a TTV viral load <4.6 log10 c/mL or above 6.6 log10 copies/mL [6].

Peripheral blood samples were collected in Streck Cell-Free DNA BCT tubes one month after the transplant, in order to determine the relative amount of circulating donor-derived cell-free DNA (dd-cfDNA). This was achieved using a targeted NGS assay that utilised 202 single nucleotide polymorphisms (AlloSeq cfDNA, CareDx, Inc., Brisbane, CA, USA) [12]. According to the manufacturer’s instructions, a cut-off of 1.0% was used as an abnormal dd-cfDNA result to define “high molecular injury” [14]. We included 42 patients in this study for whom dd-cfDNA levels and TTV viral loads were available at one-month post-transplant.

To express continuous variables, the median and interquartile range (IQR) were utilised; relative frequencies were employed to characterise categorical variables. The Mann–Whitney U test was used to compare continuous variables between dichotomous data, and the chi-squared test was used to examine the relationship between two qualitative variables. Paired TTV loads were compared using the Wilcoxon signed rank test. Univariate and multivariate regression analyses were performed to assess the variables associated with TTV load in the third month and the differences in TTV load between months 1 and 3. A *p*-value less than 0.05 was considered statistically significant. Statistical analyses were performed with SPSS version 22.0 (SPSS, Inc., Chicago, IL, USA).

## 3. Results

**Figure 1:**
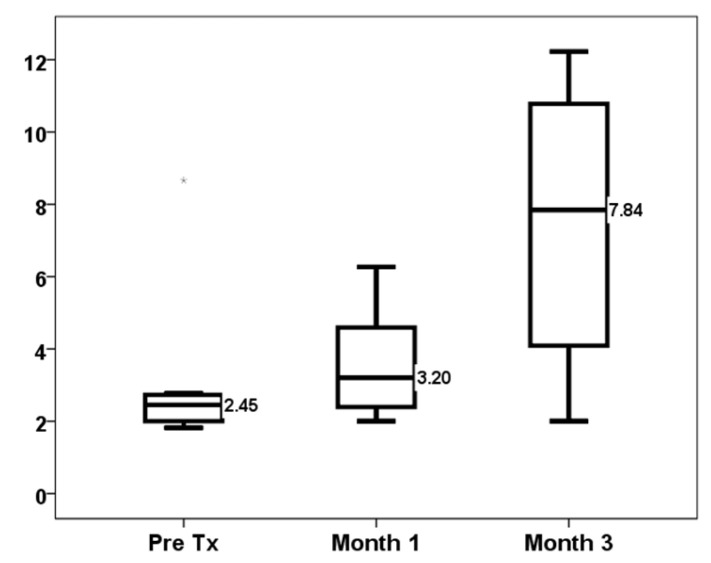
Kinetics of TTV load (log10 copies/mL).

Among 42 kidney transplant recipients with dd-cfDNA measured in the first month, we selected 11 with a high molecular injury defined by dd-cfDNA ≥ 1.0%. The main patient characteristics of these 11 patients and a comparison with the whole group are shown in Table 1. The results mentioned in the following paragraphs refer exclusively to these 11 selected patients. TTV load increased progressively from baseline (2.45, IQR 2.00–2.77 log10 copies/mL) to months 1 (3.20, IQR 2.09–5.42 log10 copies/mL, *p* = 0.008) and 3 (7.84, IQR 2.99–11.01 log10 copies/mL, *p* = 0.037) (Figure 1). There were one (9.1%) and six (54.5%) overimmunosuppressed patients in the first and third months, respectively. Conversely, eight (72.7%) and three (27.3%) patients were underimmunosuppressed in the first and third months, respectively.

There was no significant difference in TTV load at month one between patients receiving or not receiving induction therapy (2.95, IQR 2.98 log10 copies/mL vs. 3.77, IQR 3.36 log10 copies/mL, *p* = 0.376). While the differences were also not significant at month three, there was a trend in patients receiving induction therapy of showing a higher TTV load (9.23, range 6.27 log10 copies/mL vs. 2.99, range 5.84 log10 copies/mL, *p* = 0.133). Only one patient (12.5%) receiving induction was overimmunosuppressed at month one, whereas none of the patients not receiving induction were overimmunosuppressed at month one (*p* = 0.521). At month three, five (62.5%) patients treated with induction became overimmunosuppressed, while one (33.3%) patient who did not receive induction was overimmunosuppressed (*p* = 0.387). Conversely, underimmunosuppression was observed in one (33.3%) and two (25.0%) recipients at month one (*p* = 0.782) and in seven (87.5%) and one (33.3%) recipients at month three (*p* = 0.072), respectively, with and without induction therapy.

Four patients suffered AbMR and four more patients suffered different complications that could justify the high molecular injury (three patients required procedures on the graft due to hydronephrosis, one of whom suffered graft pyelonephritis caused by Klebsiella sp, and another patient presented with cytomegalovirus infection in the first month post-transplant). TTV loads in the first month were not different between patients with and without antibody-mediated rejection (2.91, IQR 4.18 log10 copies/mL vs. 3.48, IQR 1.47 log10 copies/mL, *p* = 0.788). Although 50% of underimmunosuppressed patients and none of the patients without underimmunosuppression suffered AbMR, the differences were not statistically significant (*p* = 0.125). No overimmunosuppressed patients developed AbMR, while 40% of non-overimmunosuppressed patients showed AbMR (*p* = 0.428). The AbMR rate was comparable between patients who received induction therapy and those who did not (37.5% vs. 33.3%, *p* = 0.898).

From months one to three, four (36.4%) patients developed at least one infection episode, with three (27.3%) of them opportunistic infections. Similarly, there were no differences in the risk of suffering infection (2.91, IQR 3.42 log10 copies/mL vs. 3.77, IQR 6.53 log10 copies/mL, *p* = 0.412) before month three or opportunistic infection (3.06, IQR 3.88 log10 copies/mL vs. 3.76, IQR 1.68 log10 copies/mL, *p* = 1.000) according to the TTV load in the first month. Overimmunosuppressed patients did not suffer a statistically significantly higher number of infections (30% vs. 100%, *p* = 0.165) or opportunistic infections (40% vs. 0%, *p* = 0.521). The infection rates (37.5% vs. 33.3%, *p* = 0.898) and the rates of opportunistic infection from months one to three (25.0% vs. 33.3%, *p* = 0.782) were not found to be significantly different between patients receiving and not receiving induction therapy.

**Figure 2:**
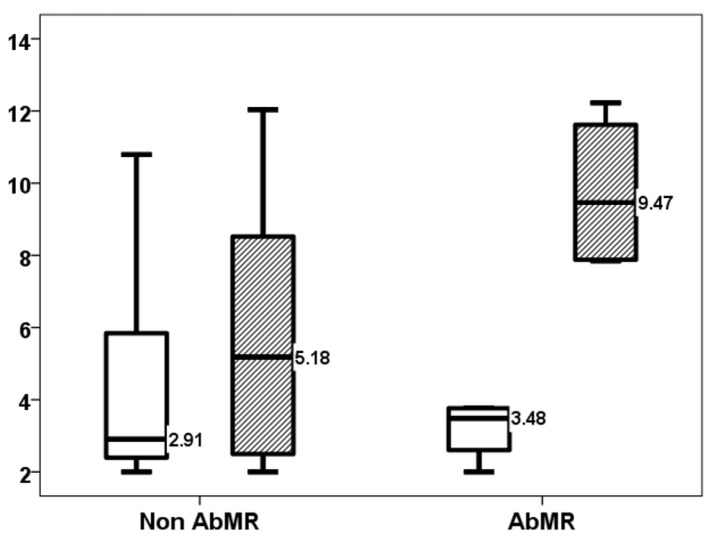
TTV load (log10 copies/mL) at 1st month (white bars) and 3rd month (stripped bars) in patients with and without AbMR.

**Figure 3:**
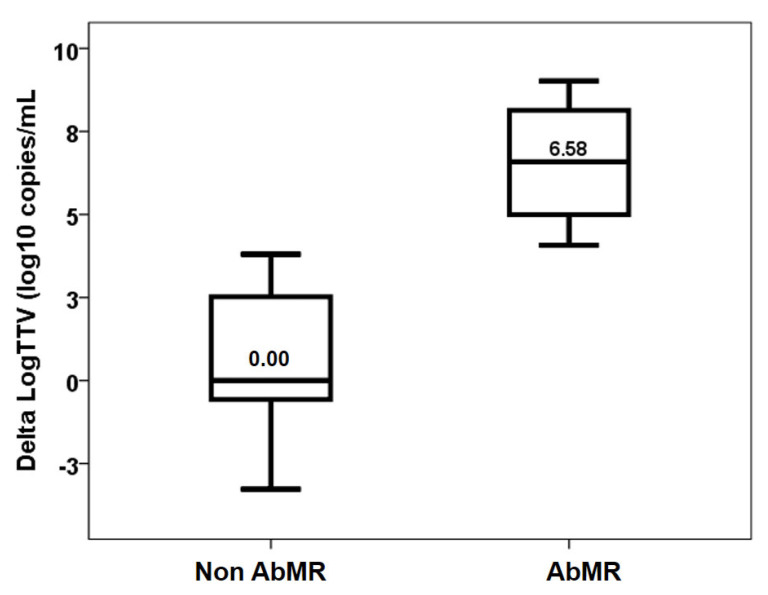
Delta LogTTV (log10 copies/mL) in patients with and without AbMR (*p* = 0.042).

**Figure 4:**
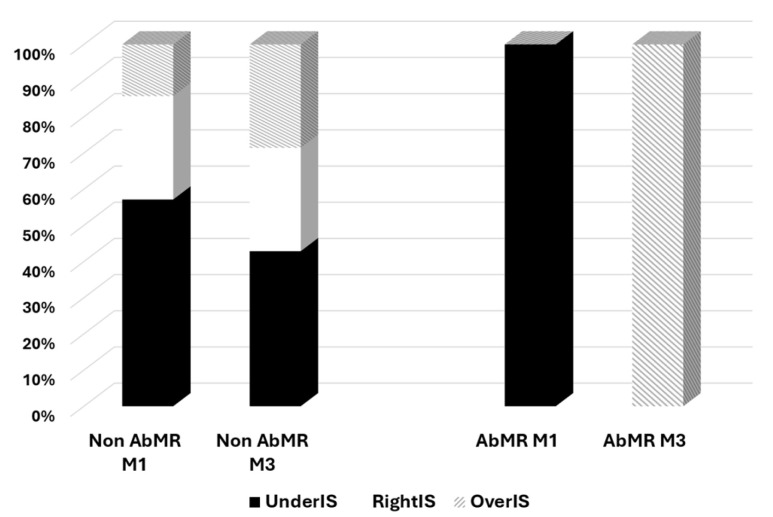
Classification according to the immunosuppressive level in patients receiving or not receiving AbMR therapy. Non AbMR = no diagnosis of antibody-mediated rejection; AbMR = antibody-mediated rejection; M1 = month 1; M3 = month 3; UnderIS = underimmunosuppressive status, OverIS = overimmunosuppressive status; RightIS = right immunosuppressive status.

As previously reported, the TTV load increased up to month three. Four patients with AbMR received specific therapy with plasmapheresis and IVIg following the recommendations [17]. TTV load in the third month showed a trend of being higher in patients who received AbMR therapy (5.18, IQR 8.55 log10 copies/mL vs. 9.47, IQR 4.06 log10 copies/mL, *p* = 0.109) (Figure 2). The difference in TTV load between months one and three was significantly higher in the AbMR-treated kidney transplant recipients (0.00, IQR 4.71 log10 copies/mL vs. +6.58, IQR 4.04 log10 copies/mL, *p* = 0.042) (Figure 3). All treated patients became overimmunosuppressed, whereas the rate of overimmunosuppression was 28.6% in non-treated patients (0.022) (Figure 4). Univariate linear regression analysis revealed a correlation between AbMR therapy and increased TTV load variations between months one and three (β 0.642, 95%CI 0.529–10.005, *p* = 0.033). To determine whether this influence could be related to the induction therapy, we performed a multivariate analysis, which revealed that AbMR therapy was associated (β 0.624, 95%CI 1.120–9.110, *p* = 0.01) with a higher increase in TTV load between month one and three, independently of induction therapy (β 0.481, 95%CI −0.059–8.571, *p* = 0.053).

**Figure 5:**
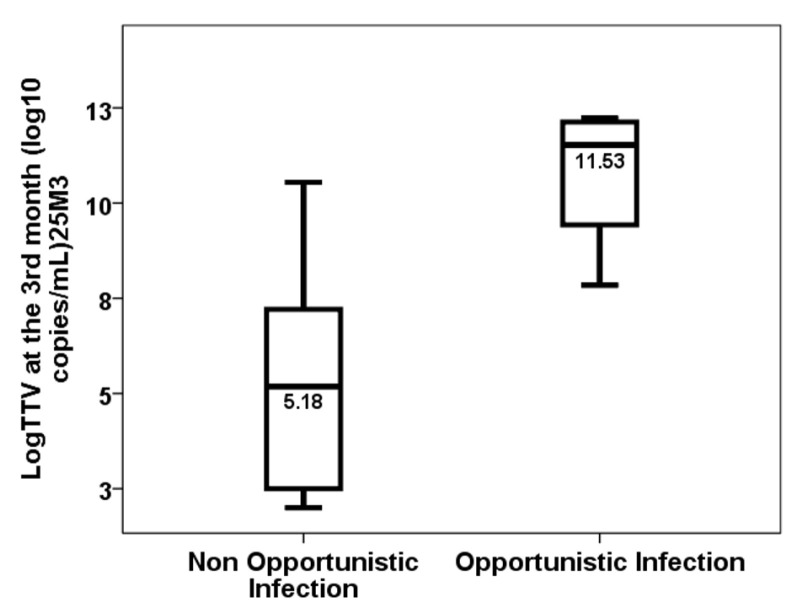
LogTTV (log10 copies/mL) in the 3rd month in patients with and without opportunistic infection between months 3 and 6.

Eight patients (72.7%) developed at least one infection episode and four developed opportunistic infections (36.4%) between months three and six. TTV load in the third month was not significantly different between patients without and with infection between months three and six (2.99, IQR 4.5 log10 copies/mL vs. 9.23, IQR 5.93 log10 copies/mL, *p* = 0.085). Those with opportunistic infections showed higher TTV loads in the third month (5.18, IQR 5.92 log10 copies/mL vs. 11.53, IQR 3.54 log10 copies/mL, *p* = 0.024) (Figure 5). Overimmunosuppressed patients suffered a significantly higher rate of infections (40.0% vs. 100.0%, *p* = 0.026) and opportunistic infections (0.0% vs. 66.7%, *p* = 0.022) between months three and six. The infection rates (87.5% vs. 33.3%, *p* = 0.072) and the rates of opportunistic infection between months three and six (37.5% vs. 33.3%, *p* = 0.898) were not significantly different in patients receiving induction therapy compared to those not receiving it. Univariate linear regression analysis showed that TTV load in the third month was higher in patients who developed opportunistic infections between the third and sixth months (β 3.021, 95%CI 1.374–9.574, *p* = 0.014). After multivariate analysis, kidney transplant recipients who developed opportunistic infections between months three and six showed higher TTV loads (β 3.726, 95%CI 2.033–8.635, *p* = 0.006) independently of induction therapy (β 2.539, 95%CI 0.360–7.491, *p* = 0.035).

## 4. Discussion

The development of new monitoring methods for solid organ transplants can facilitate their follow-up and improve long-term outcomes by allowing earlier and minimally invasive detection of both rejection and infection risk. Many of these new markers offer complementary information, and their combined use could be of great help in addition to conventional monitoring [18]. For example, a higher percentage of donor-derived cell-free DNA circulating in the recipient’s blood has been associated with a higher risk of current or future rejection, especially AbMR. It can also be used as a marker of response to rejection treatment and of the risk of infections in the renal graft [9,10,11,12,13,14,19,20,21,22]. On the other hand, the measurement of TTV viral load allows the global state of immunosuppression of the patient to be assessed and thus the identification of whether they are at risk of developing rejection if they are underimmunosuppressed, or infection if they are overimmunosuppressed [6,7,8]. In this regard, we carried out for the first time a dual monitoring using dd-cfDNA and TTV viral load in kidney transplant recipients with the aim of identifying which patients were experiencing molecular damage in the kidney graft and differentiating whether the cause of the damage was infectious or secondary to the alloimmune response, based on the estimated global level of immunosuppression using the TTV viral load. Interestingly, we found that patients with high molecular damage who were experiencing rejection tended to have lower TTV viral load levels, although the small number of patients studied limited the degree to which the results could reach statistical significance. Similarly, only patients with low TTV viral loads, labelled as underimmunosuppressed, experienced rejection, while none of the overimmunosuppressed patients did. Conversely, the only patient with cytomegalovirus infection was the most overimmunosuppressed patient (TTV load of 10.79 log10 copies/mL). These findings suggest that the combined measurement of dd-cfDNA and TTV viral load could non-invasively identify which patients with molecular damage were underimmunosuppressed and at risk of rejection or overimmunosuppressed and at risk of graft infection. However, our study is underpowered to confirm this hypothesis, so we suggest that new studies including more patients using this dual monitoring should be carried out.

Since the viral load of TTV is a measure of the state of immunosuppression, it is of utmost interest to know what the relationship is between that and the immunosuppressive drugs used in solid organ transplantation. The results of previous studies have provided partially contradictory results. In relation to calcineurin inhibitors (CNIs), some studies detected that patients with higher levels presented a higher viral load [23,24]. However, this finding was not confirmed in other studies [25,26,27,28,29,30,31,32]. In our population of renal transplant recipients, we previously demonstrated the absence of a relationship between TTV viral load and trough levels of tacrolimus, cumulative exposure measured by the mean value of trough levels, the concentration/dose ratio (C/D) of tacrolimus, and two exposure variability markers: the coefficient of variability and time in the therapeutic range [15]. We also did not observe that TTV viral load was related to trough levels of mycophenolic acid or its area under the curve [15]. Rather than being related to levels maintained over time, the viral load seems to be related to changes in immunosuppressants. On the one hand, Regele et al. observed that the reduction in the dose and levels of tacrolimus was associated with a decrease in TTV viral load two months after the dose change, although the increase in dose and levels was not significantly associated with an increase in viral load [33]. On the other hand, Benning et al. and Regele et al. have reported that TTV viral load decreases after the withdrawal of mycophenolic acid [34,35]. It is also not well-known if the increased immunosuppression associated with the treatment of acute rejection modifies the TTV viral load. Reineke et al. have reported that treatment with high-dose corticosteroid pulse therapy as anti-rejection therapy significantly increased the TTV viral load from before the biopsy (median 4.07 log10 copies/mL, IQR 3.22–5.01 log10 copies/mL) to days 30 (median 4.88 log10 copies/mL, IQR 4.06–6.13 log10 copies/mL, *p* < 0.005) and 90 post-treatment (median 5.26 log10 copies/mL, IQR 4.28–7.57 log10 copies/mL, *p* < 0.001) in 31 patients [36]. Our results reflect the evolution of patients treated for antibody-mediated rejection for the first time. The difference in TTV viral load was +6.58 log10 copies/mL in treated patients compared to untreated patients. Although the number of patients was low, our study suggests that treatment with plasmapheresis and IVIg increases the TTV viral load more intensely than treatment with high-dose corticosteroids. In fact, all patients treated for AbMR became overimmunosuppressed and, therefore, at a high risk of infection. This finding should be confirmed in further studies.

In the field of immunosuppressive therapy, the choice of induction therapy has been shown to have a significant impact on the outcome of a kidney transplant. Induction therapy with thymoglobulin has been shown to reduce the risk of acute rejection and increase the risk of developing opportunistic infections, while Basiliximab reduces the occurrence of rejection in the first year [37,38]. In this regard, it is essential to understand how induction therapy influences the TTV viral load and whether it alters the relationship between viral load and both rejection and the risk of infections of significant concern. Various studies have linked receiving induction therapy, or specifically thymoglobulin, with a higher TTV viral load in the first months after solid organ transplantation [15,25,27,32,39,40,41,42]. The impairment of T-cell-mediated immunity caused by induction therapy, especially thymoglobulin, appears to favour TTV replication; however, due to the low number of cases, in our study we only found a non-significant trend toward higher viral loads in patients who had received induction. Nevertheless, it was observed that the TTV load and its growth from months one to three were higher in patients who suffered from opportunistic infections beyond the third month and in those who had received therapy for AbMR, respectively, regardless of the use of induction.

Although we did not find a significant relationship between a high TTV load in the first month and the risk of infection, our results showed that the TTV load in the third month was associated with the development of infection. This finding has previously been reported by most, but not all, studies conducted in kidney transplant recipients [15,39,40,41,43,44,45,46,47]. A meta-analysis including seven studies analysing the performance of the TTV load for infection discrimination in kidney transplant recipients reported that the pooled area under the receiver operating characteristics curve was 0.68, with the TTV load being linearly associated with infection [8]. The Vienna group reported that the TTV load was already lower 1–3 months (27–77 days) before the development of infection [43,44]. In contrast, the absence of a relationship between the TTV load at month one and the development of infections in our study could be due not only to the low number of patients included but also to the low incidence of infectious events between the first and third months.

The present study demonstrated the usefulness of measuring the TTV load to estimate the immunosuppressive status, even in a specific high-risk group of patients with high molecular injury and a high AbMR rate. The study also showed that ‘TTV load-defined’ overimmunosuppressed kidney transplant recipients had both very high infection and opportunistic infection rates. Consequently, we support the utilisation of the cut-off values proposed by the Vienna group to monitor the immunosuppressive status of kidney transplant recipients at risk, a practice that should be implemented from the first month up to the 12th month of the post-transplantation period [6]. Interestingly, the Lyon group has reported different TTV load thresholds (3.75–5.1 log10 copies/mL) in kidney transplant recipients beyond the first year after transplantation, which were associated with a reduced complication risk. In this study, a TTV load above the upper cut-off was the sole variable independently related (HR 9.41, 95%CI 2.52–35.20) to the risk of infection or cancer as an overimmunosuppression biomarker [48]. We can argue about the usefulness of knowing the net state of immunosuppression in a kidney transplant recipient who has been treated for a previous rejection episode. It is evident that the patient is likely to become overimmunosuppressed following rejection therapy, or alternatively, the rejection-related underimmunosuppressive status is reversed. However, the identification of the overimmunosuppressive status through measuring the TTV load in high-risk patients would facilitate the design of surveillance strategies to detect infection earlier or modify prophylactic therapy. For instance, this could involve prolonging cytomegalovirus prophylaxis beyond the third month only in these patients. These suggestions require confirmation through specifically designed studies.

The limitations of the present study are evident. The sample size is small, the follow-up period is short, and the study is single-centre, which limits the generalisability of the results without larger multicentre prospective studies. However, the study is significant because, for the first time, it performs a joint prospective analysis of two relevant biomarkers that provide complementary information for the follow-up of solid organ transplants.

In conclusion, we have conducted a pioneering study in which we have utilised a dual monitoring approach in kidney transplant recipients, employing biomarkers that offer complementary information. This approach has the potential to enhance post-transplant monitoring. Our methodology involved the use of donor-derived cell-free DNA to identify recipients with greater molecular injury, and TTV load as a metric to assess the overall immunosuppressive status. The results of this study indicated that patients exhibiting molecular damage secondary to rejection tended to be underimmunosuppressed, as indicated by a low TTV load. Following treatment for antibody-mediated rejection, there was a notable increase in TTV load, resulting in a transition from under- to overimmunosuppressed status for all patients. Furthermore, a high TTV load during the third month post-transplant was found to be associated with an elevated risk of opportunistic infections. Of interest, these relationships were independent of exposure to induction therapy. The measurement of TTV load in high-risk patients facilitates the identification of those who are overimmunosuppressed and thus have an increased susceptibility to infection. It is hypothesised that these patients would benefit from the implementation of more rigorous strategies for the early detection of opportunistic infections or alterations in prophylaxis regimens. However, the efficacy of these approaches must be evaluated through the execution of meticulously designed studies.

## Figures and Tables

**Table 1:** Main patient characteristics.

Number of Patients	11	31	*p*
Recipient age (years)	60.0 [48.0–73.0]	59.0 [47.0–66.0]	0.572
Recipient sex (male)	63.6%	80.6%	0.255
Diabetic nephropathy	18.2%	25.8%	0.610
Time in renal replacement therapy (months)	24.5 [10.5–108.7]	10.7 [0.0–32.6]	0.062
Retransplant	36.4%	12.9%	0.089
Pre-emptive transplantation	18.2%	22.6%	0.760
Donor age (years)	61.0 [47.0–65.0]	58.0 [47.0–65.0]	0.910
Antigen HLA Class-I and -II mismatches	8.0 [7.0–9.0]	7.0 [5.0–9.0]	0.233
Cold ischaemia time (h)	20.0 [18.0–24.0]	22.0 [17.0–24.0]	0.822
Induction	72.7%	64.5%	0.620
Thymoglobulin (%)	45.5%	16.1%	0.050
Basiliximab (%)	27.3%	48.4%	0.224
Delayed graft function (%)	36.4%	22.6%	0.372
1-month AbMR	36.4%	3.2%	0.004
Infection from months 1 to 3	36.4%	41.9%	0.746
Opportunistic infection from months 1 to 3	27.3%	12.9%	0.272
First-month eGFR (mL/min/1.73 m^2^)	43.0 [28.0–54.0]	51.0 [42.0–72.0]	0.138
1-year eGFR (mL/min/1.73 m^2^)	45.0 [38.0–49.0]	50.0 [38.0–71.8]	0.514
First-month mean Tacrolimus trough level (ng/mL)	12.5 [11.4–14.3]	12.3 [11.2–14.2]	0.822
Tacrolimus trough level at month 1 (ng/mL)	13.0 [10.0–15.0]	12.0 [9.0–14.0]	0.233
Mycophenolic acid trough level at month 1 (ng/mL)	2.0 [1.8–4.0]	2.0 [1.0–3.0]	0.569
First-month TTV load (log10 copies/mL)	3.20 [2.09–5.41]	2.23 [1.18–3.28]	0.117
Third-month TTV load (log10 copies/mL)	7.84 [3.00–11.01]	5.72 [2.85–8.59]	0.295

## Data Availability

The data presented in this study are available on request from the corresponding author.

## Abbreviations

The following abbreviations are used in this manuscript:

AbMR	antibody-mediated rejection
CMV	cytomegalovirus
dd-cfDNA	donor-derived circulating free DNA
EBV	Epstein–Barr virus
HBV	Hepatitis B virus
HCV	Hepatitis C virus
HSV	Herpes simplex virus
IQR	interquartile range
MPA	mycophenolic acid
KTx	kidney transplant recipients
TTV-load	Torque Teno virus load
VZV	Varicella zoster virus
